# A *Verticillium dahliae* Pectate Lyase Induces Plant Immune Responses and Contributes to Virulence

**DOI:** 10.3389/fpls.2018.01271

**Published:** 2018-09-13

**Authors:** Yuankun Yang, Yi Zhang, Beibei Li, Xiufen Yang, Yijie Dong, Dewen Qiu

**Affiliations:** The State Key Laboratory for Biology of Plant Diseases and Insect Pests, Institute of Plant Protection, Chinese Academy of Agricultural Sciences, Beijing, China

**Keywords:** *Verticillium dahliae*, pectate lyase, elicitor, plant immunity, virulence

## Abstract

*Verticillium dahliae* is a wide-host-range fungal pathogen that causes soil-borne disease in hundreds of dicotyledonous hosts. In search of *V. dahliae* Vd991 cell death-inducing proteins, we identified a pectate lyase (VdPEL1) that exhibited pectin hydrolytic activity, which could induce strong cell death in several plants. Purified VdPEL1 triggered defense responses and conferred resistance to *Botrytis cinerea* and *V. dahliae* in tobacco and cotton plants. Our results demonstrated that the mutant VdPEL1^rec^ lacking the enzymatic activity lacked functions to induce both cell death and plant resistance, implying that the enzymatic activity was necessary. In addition, *VdPEL1* was strongly induced in *V. dahliae* infected *Nicotiana benthamiana* and cotton roots, and *VdPEL1* deletion strains severely compromised the virulence of *V. dahliae*. Our data suggested that VdPEL1 contributed to *V. dahliae* virulence and induced plant defense responses. These findings provide a new insight for the function of pectate lyase in the host–pathogen interaction.

## Introduction

Plants are exposed to a multitude of pathogens that cause massive yield and quality losses annually. To ward off invading microorganisms, plants have evolved elaborate systems to provide better immunity against pathogens. Plants utilize pattern recognition receptors (PRRs) to perceive pathogen-associated molecular patterns (PAMPs) or damage-associated molecular patterns (DAMPs) and rapidly activate PAMP-triggered immunity (PTI). This recognition initiates a cascade of signaling events resulting in induction of a battery of immune responses, including Ca^2+^ influx, the burst of reactive oxygen species (ROS), the accumulation of defense hormones, the expression of defense-related genes and callose deposition (Boller and Felix, 2009; Couto and Zipfel, 2016). In turn, during the coevolution of hosts and microbes, pathogens also employ numerous effectors to interfere with PTI and establish successful infection, which is regarded as effector-triggered susceptibility (ETS) (Chisholm et al., 2006; Jones and Dangl, 2006; Saijo et al., 2017). As an adaption to ETS, the effectors are recognized by the resistance (R) proteins, and subsequently lead to robust immunity termed effector-triggered immunity (ETI) (Houterman et al., 2008; Stergiopoulos and de Wit, 2009). Generally, ETI is associated with stronger immune responses, such as the hypersensitive response (HR). Plant immune responses initiate from local tissues located at the site of the infection and subsequently extend to other non-infected tissues by long-distance intercellular communications, generating a systemic acquired resistance (SAR) that is effective against a broad spectrum of pathogens in the whole plant (Kulye et al., 2012).

The plant cell wall is the first barrier, which provides mechanical strength and rigidity to protect against pathogenic infection. Correspondingly, pathogens secrete numerous of cell wall-degrading enzymes (CWDEs) to permit them to invade plant tissue and supply themselves with nutrients (Kikot et al., 2009; Klöckner et al., 2016). On the one hand, CWDEs serve as virulence factor in pathogens and play an essential role in infection process. Previous studies suggested that the deletion of the Xylanase *Xyn11A* gene caused a marked effect on the ability of *Botrytis cinerea* to infect tomato leaves and grape berries (Brito et al., 2006). And an endoxylanase named xynB contributed to virulence in *Xanthomonas oryzae pv. oryzae* (Pandey and Sonti, 2010). Alternatively, plants can also sense CWDEs or self-molecules (released from plant cell wall polysaccharides) as inducers of immune responses (Misas-Villamil and van der Hoorn, 2008). Recently, it was widely reported that glycoside hydrolase 12 (GH12) proteins, VdEG1 and VdEG3 from *Verticillium dahliae*, XEG1 from *Phytophthora sojae* and BcXYG1, a secreted xyloglucanase from *B. cinerea* contributed to virulence and simultaneously triggered plant immunity as PAMPs (Gui Y.-J. et al., 2017; Zhu et al., 2017a,b). Oligogalacturonides (OGs) were regarded as DAMPs to activate the plant immune responses (De Lorenzo et al., 2011). The enzymatic activity of VdCUT11(a cutinase from *V. dahliae*) was required for activating immune responses in *Nicotiana benthamiana*, implying that the cutinase degradation products might induce the plant resistance (Gui Y. et al., 2017).

Pectin exists widely in the plant cell walls and cell lining and maintains wall integrity and cell–cell cohesion. Due to the complexity of this highly branched polysaccharide, the degradation of pectin requires a variety of enzymes, such as pectin lyases, pectate disaccharide-lyases, and pectate lyases. Among these, pectate lyases have received the most attention. Pectate lyases randomly cleave α-1,4-polygalacturonic acid via a β-elimination reaction. They can also macerate and disassemble the plant cell wall and tissues in a manner similar to that occurring in fungal diseases (Collmer, 1986). For instance, the deletion of the pectate lyase gene *CcpelA* and *PelB* in *Colletotrichum coccodes*, resulted in attenuated virulence on green tomato fruit and reduced susceptibility on avocado (*Persea americana*) fruit, respectively (Yakoby et al., 2001; Ben-Daniel et al., 2011). The deletion of the pectate lyase genes *BcPG1* and *BcPG2* also reduced virulence in *B. cinerea* (Have et al., 1998; Kars et al., 2005). However, pectic enzymes can also elicit plant defense responses through direct or indirect ways. For instance, a pectate lyase, from *Erwinia carotovora* bacteria, induced defense responses against Erwinia soft rot in potato plants (Wegener, 2002).T4BcPG1, an endopolygalacturonase from *B. cinerea*, activated grapevine defense reactions (Poinssot et al., 2003).

*Verticillium dahliae* is a wide-host-range pathogen that can infect a large number of dicotyledonous hosts, including ecologically important plants and many high-value crops worldwide such as cotton, tobacco, potato, and tomato (Fradin and Thomma, 2006; Klosterman et al., 2009; Inderbitzin et al., 2014). It infects plants primarily through the formation of hyphae, which can puncture the plant root surface and colonize in xylem vessels (Zhao et al., 2014). It was demonstrated that *V. dahliae* secrets a large amount of CWDEs, including 13 pectate lyases. However, the specific roles of the pectate lyases in *V. dahliae* remain largely unknown.

The main objectives of the current study were to: (1) isolate and investigate cell death-inducing proteins in *V. dahliae* Vd991; (2) determine whether VdPEL1 is secreted into the apoplast in order to induce cell death; (3) examine the relationship between the enzymatic activity and cell death-inducing activity; and (4) investigate the function of VdPEL1 in immune responses and virulence.

## Materials and Methods

### Fungal Cultures, Plants Grown

The *V. dahliae* strains, including Vd991 wild type strains, targeted deletion strains and complementary transformants, were maintained and cultured on PDA media or in liquid Czapek media for 7 days at 25°C. *B. cinerea* strain BC-98 and *Agrobacterium tumefaciens* AGL-1 were grown on PDA media at 25°C and LB (Kan and Rif) medium at 28°C, respectively. Cotton (*Gossypium hirsutum cv. Junmian 1*) and *N. benthamiana* were grown at 23 and 27°C, respectively, in a greenhouse with a day/night period of 14/10 h.

### Separation, Purification, and Characterization of Proteins Secreted From *V. dahliae*

To produce large amounts of secreted proteins, Vd991 was grown in Czapek media at 25°C for 14 days, shaken at 150 rpm (Bailey, 1996), and then filtered through two layers of filter paper. The supernatants were collected and precipitated with 70% (NH)_4_SO_4_ overnight at 4°C. The precipitate was collected by centrifugation at 15,000 ×*g* for 10 min at 4°C, and then resuspended in 10 mM Tris-HCl (pH 7.5) and 1 mM EDTA (TE). Crude protein was further purified by ion-exchange chromatography and eluted with a linear sodium chloride gradient from 0.0 to 1.0 M in TE. Fractions corresponding to absorbance peaks were desalted and concentrated using a 10-kDa ultrafiltration device and tested their ability to induce cell death activity. The fraction with cell death activity was excised from the SDS-PAGE gel and identified using mass spectrometry (MS) analysis (Beijing Protein Innovation, Beijing, China) as described (Heese et al., 2007). The tandem MS + MS/MS data were automatically analyzed using the Mascot search engine (Matrix Science, London, United Kingdom).

### Cloning, Expression, and Purification of Recombinant Protein

VdPEL1 fragment (amplified with primers VdPEL1F/VdPEL1R; **Supplementary Table S2**) without the predicted signal peptides and stop codons was inserted into the pPICZαA plasmid at the *Bam*HI and *Eco*RI sites (Ma et al., 2015). The recombinant plasmid pPICZαA-VdPEL1 was linearized with *Pme*I and transformed into *Pichia pastoris* KM71H for expression. The transformed yeasts were grown and induced in BMGY (buffered glycerol-complex medium) and BMMY (buffered methanol-complex medium), respectively (Easy Select Pichia Expression Kit; Invitrogen). The supernatant was collected (3,000 ×*g* for 10 min at 4°C) and then purified using nickel affinity chromatography (Ma et al., 2015; Zhang et al., 2017). The purified VdPEL1 or VdPEL1^rec^ were kept in protein buffer (20 mM Tris, pH 8.0) and further detected via SDS-PAGE and Western blotting. The concentration of the purified protein was measured using an Easy II Protein Quantitative Kit (BCA) and the protein was then stored at -80°C.

### Site-Directed Mutagenesis

To examine the relationship between the enzymatic activity and cell death-inducing activity of VdPEL1, we constructed a VdPEL1^rec^ mutant with loss of the enzymatic activity. According to multiple sequence alignment, we found that Asp-125 and Asp-147 residues might be the critical catalytic sites of VdPEL1. Two Asp residues of VdPEL1 were replaced by Ala residues using the Quick Change™ Site-Directed Mutagenesis Kit (Stratagene, United States) to cause the loss of pectate lyase activity of VdPEL1. Primers used in these constructions are listed in **Supplementary Table S2**. The VdPEL1^rec^ mutant was confirmed by sequence alignment and the enzyme assays.

### Enzyme Activity Assays

Hydrolase activity was measured using the 2-cyanoacetamide spectrophotometric method (Bach and Schollmeyer, 1992). The purified VdPEL1 or VdPEL1^rec^ (500 ng) and substrate (2.5% polygalacturonic acid) were co-incubated at 30°C in a medium buffered with MES or Tris 10 mM at indicated pH (total volume: 350 μl). Fifteen minutes later, all samples were incubated for 10 min at 100°C to terminate the assays, and the reduced sugars released by VdPEL1 or VdPEL1^rec^ were measured at 274 nm using a spectrophotometer. The reduced sugars were quantified using a standard calibration curve obtained with polygalacturonic acid. The experiment was repeated three times.

### Immunoblot Analysis

To confirm whether VdPEL1 was secreted into the apoplast, transient expression in *N. benthamiana* was performed. Three sequences, encoding VdPEL1 protein with the putative signal peptide, VdPEL1 protein without the putative signal peptide and PR1 SP-VdPEL1^21-255^ protein replaced the signal peptide from pathogenesis-related protein 1 (PR1), were amplified with primers VdPEL1-T-F/VdPEL1-T-R, VdPEL121-255-F/VdPEL121-255-R, and PR1 SP-VdPEL121-255-F/PR1 SP-VdPEL121-255-R, respectively (**Supplementary Table S2**). Three sequences were cloned into the pYBA1132 vector which contained a C-terminal GFP tag at the *Xba*I and *Bam*HI sites (Yan et al., 2012). To confirm whether the enzymatic activity of VdPEL1 was related to the cell death-inducing activity, the sequences encoding the mature VdPEL1 protein and site-directed mutagenized VdPEL1^rec^ with the putative signal peptide (amplified with primers VdPEL1F/VdPEL1R and VdPEL1rec F/VdPEL1rec R, **Supplementary Table S2**) were cloned into pYBA1132 vector at the *Xba*I and *Bam*HI sites, and then transformed into the *A. tumefaciens* strain GV3101. Agroinfiltration assays were performed on *N. benthamiana* plants. To determine whether fusion proteins were expressed, plant total protein extractions, and immunoblots were assessed as previously described (Yu et al., 2012). All the proteins were analyzed via immunoblots using anti-GFP-tag primary monoclonal antibody. The blots were visualized using the Odyssey^®^ LI-COR Imaging System. Rubisco was used to confirm the equal protein loading.

### Elicitor Activity and Systemic Resistance Assays

To detect the induction of cell-death, 300 nM purified VdPEL1 or VdPEL1^rec^, PEVC and BSA were injected into leaves of 4-week-old *N. benthamiana* plants and 2-week-old plants of cotton, tomato, and soybean with an injector. These plants were kept in a glasshouse with a day/night period of 14/10 h, and cell-death responses were investigated after 48 h of treatment with the recombinant proteins. The accumulation of ROS in the plant leaves was stained by using 3′3-diaminobenzidine (DAB) solution as described previously (Bindschedler et al., 2006). To visualize callose deposition, 4-week-old *N. benthamiana* leaves were treated with 300 nM recombinant proteins and stained with aniline blue at 24 h post-treatment, as described previously (Chen et al., 2012). To assay electrolyte leakage, 4-week-old *N. benthamiana* leaves were infiltrated with 300 nM purified VdPEL1 or VdPEL1^rec^ and PEVC. The corresponding *N. benthamiana* leaves at different time points were harvested and submerged in sterile water at 4°C. Ion conductivity was measured using a conductivity meter.

The purified VdPEL1 or VdPEL1^rec^ and PEVC were individually syringe-infiltrated into 4-week-old *N. benthamiana* leaves. A total of 5 μl of 2 × 10^6^ conidia/ml *B. cinerea* or 1 × 10^6^ conidia/ml *V. dahliae* were placed on the infiltrated area or inoculated by the root-dip method, respectively. The inoculated plants were placed in a greenhouse with a day/night period of 14/10 h. The lesion development of *B. cinerea* on the *N. benthamiana* leaves was evaluated at 2 days post-inoculation by determining the average lesion diameter. In addition, disease symptoms were observed at 12 days *V. dahliae* post-inoculation on *N. benthamiana*. All the experiments were performed three times.

### Generation of *VdPEL1* Deletion and Complementary Mutants

The wild-type *VdPEL1* gene and 500 bp flanking sequences of the target gene were amplified from the *V. dahliae* Vd991 genomic DNA (gDNA). Two flanking sequences of the target gene and a hygromycin resistance cassette were constructed into a fusion fragment using a nested PCR reaction, which was subsequently introduced into the binary vector pGKO2-Gateway. To generated complementary transformants, the donor vector pCT-HN containing *VdPEL1* gene was integrated into the mutant transformants using an *Agrobacterium-*mediated transformation method (Liu et al., 2013). All the mutants were identified using PCR with the corresponding primers.

### RNA Extraction and qRT-PCR

To measure the expression of *VdPEL1* gene during infection, 4-week-old *N. benthamiana* plants or 2-week-old cotton seedlings were inoculated with 1 × 10^6^ *V. dahliae* conidia/ml or 5 × 10^6^ conidia/ml by the root-dip method, respectively. We selected 10 indicated time points during different stages of post-inoculation to determine expression patterns of *VdPEL1* using quantitative PCR (qPCR). All the samples were obtained at the time points indicated and stored at –80°C. Total RNA from *V. dahliae* infected plants was extracted with the E.Z.N.A.^®^ Total Fungal RNA Kit I. To measure the expression of defense-related genes, the leaves of 4-week-old *N. benthamiana* plants were treated with 300 nM purified VdPEL1 or VdPEL1^rec^ and PEVC. The leaves were obtained at the time points indicated, immediately frozen in liquid nitrogen, and stored at -80°C. A EasyPure Plant RNA Kit (TransGen Biotech) was used to extract the total plant RNA. The gDNA was digested by the DNase I (TransGen Biotech). And the gDNA remover was added when the cDNA was synthesized. To further investigate whether or not the gDNA was absolutely removed, the gene encoding the actin (GenBank: X63603.1) in tobacco and *β-tubulin* (VDAG_10074) in *V. dahliae* were detected by PCR, respectively.

qRT-PCR was performed using a TransStart Green qPCR SuperMix UDG according to the manufacturer’s instructions (TransGen Biotech). qRT-PCR was performed under the following conditions: an initial 95°C denaturation step for 10 min followed by 40 cycles of 95°C for 15 s and 60°C for 1 min. The cotton 18S gene, *N. benthamiana EF-1a* and *β-tubulin* (VDAG_10074) of *V. dahliae* were used as endogenous plant controls and used to quantify fungal colonization, respectively. All primers are listed in **Supplementary Table S2**. The relative transcript levels among various samples were determined using the 2^-ΔΔCT^ method with three independent determinations (Livak and Schmittgen, 2001).

### Pathogenicity Assays

To test whether VdPEL1 functioned as a virulence factor of *V. dahliae*, the wild-type strain and derived mutants, including the *VdPEL1* deletion and complementary mutants were used in this study. Four-week-old *N. benthamiana* plants or 2-week-old cotton seedlings were inoculated with 1 × 10^6^ conidia/ml or 5 × 10^6^ conidia/ml by the root-dip method, respectively (Zhou et al., 2013). After 21 days post-inoculation on cotton or 14 days post-inoculation on *N. benthamiana*, disease symptom and fungal biomass was determined as previously described (Santhanam et al., 2013). Discoloration in vascular tissues was observed by cutting root longitudinal sections at 3 weeks after inoculation. Real-time qPCR was performed using a qPCR SYBR premix Ex Taq II kit (TaKaRa, Kyoto, Japan). *t*-tests were performed to determine statistical significance at *p*-values <0.05 between two treatments groups.

### Statistical Analysis

All the experiments and data presented here were performed at least three repeats. The data are presented as the mean and standard deviations. Statistical Analysis System (SAS) software was used to perform the statistical analysis via Student’s *t*-test.

## Results

### Identification, Purification, and Characterization of VdPEL1

**FIGURE 1:**
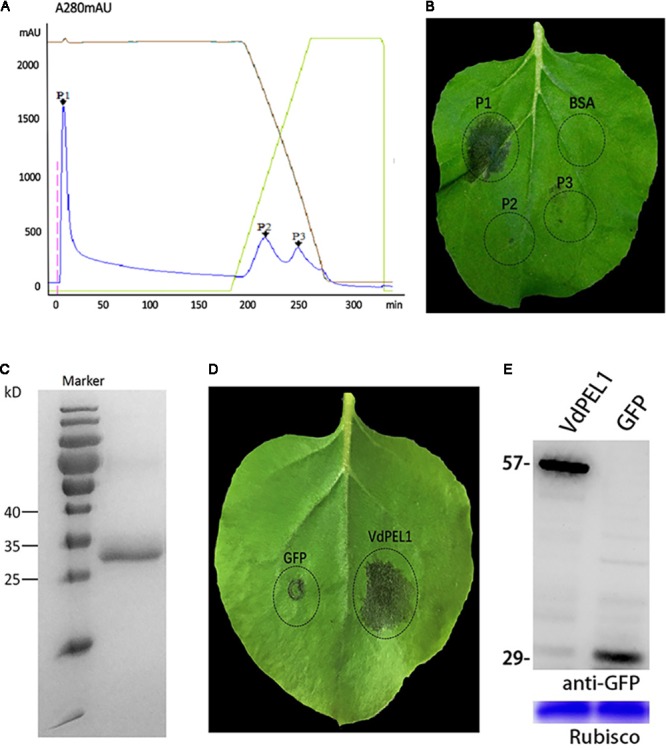
VdPEL1 purified from *V. dahliae* culture supernatants induces cell death in *N. benthamiana*. **(A)** Ion exchange chromatography of supernatant proteins, resulting in three peaks. **(B)** Two-month-old *N. benthamiana* leaves were infiltrated with fractions from the peaks or a BSA control. **(C)** SDS-PAGE analysis of peak A proteins showed a single band with a molecular mass between 25 and 35 kDa. **(D)** Transient expression of VdPEL1 in *N. benthamiana* leaves 5 days after inoculation with *Agrobacterium* strains. **(E)** Immunoblot analysis of proteins from *N. benthamiana* leaves transiently expressing GFP and VdPEL1.

To identify the defense response proteins from *V. dahliae*, the induction of cell death in *N. benthamiana* leaves was used as an index to fractionate proteins from culture filtrates via anion exchange chromatography. Due to different affinities for chromatographic column, fractionation of a 70% ammonium sulfate precipitate generated three primary peaks (**Figure 1A**). Fractions corresponding to peak 1 could induce cell death in *N. benthamiana* leaves (**Figure 1B**). Further fractionate and purify, SDS-PAGE analysis showed a single obvious band between 25 and 35 kDa (**Figure 1C**). To identify amino acid sequence of the peak 1 band, tryptic digestion was performed, and the peptides generated were analyzed by liquid chromatography-MS. Many peptides with high credibility were co-matched a protein encoded by the gene (Gene ID: 20707618) (**Supplementary Figure S1**). The bioinformatics analysis found that the gene encodes a pectate lyase in *V. dahliae* Vd991, and we designated this protein as VdPEL1. Transient expression of VdPEL1 in *N. benthamiana* leaves showed that the protein triggers cell death 5 days after infiltration (**Figure 1D**). Immunoblot analysis using a-GFP antibody detected the expression of VdPEL1 in the leaves (**Figure 1E**).

### Nucleotide Sequence Analysis of VdPEL1

**FIGURE 2:**
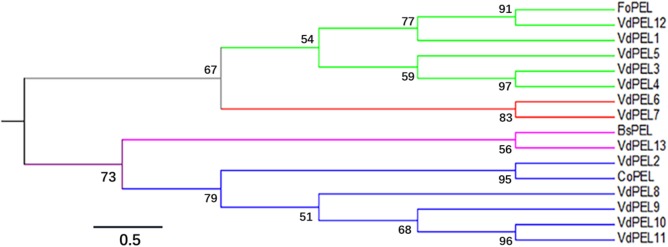
Phylogenetic relationships between the *V. dahliae* pectate lyase family members and pectate lyase from other fungi. The phylogeny was constructed by Mega 6.0 using maximum-likelihood (parameters: 1,000 bootstraps, Jones-Taylor-Thornton model). Branch colors indicated the four groups of pectate lyase family members in *V. dahliae*. The sequence data of all the proteins can be found in the GenBank/EMBL data libraries under the accession numbers: VdPEL1 (EGY15301.1), VdPEL2 (EGY22111.1), VdPEL3 (EGY18351.1), VdPEL4 (EGY13381.1), VdPEL5 (EGY19202.1), VdPEL6 (XP_009649735.1), VdPEL7 (EGY16604.1), VdPEL8 (EGY14058.1), VdPEL9 (EGY16402.1), VdPEL10 (EGY14238.1), VdPEL11 (XP_009651657.1), VdPEL12 (PAU54057.1), VdPEL13 (PAU53142.1), FoPEL (BAC74094), BsPEL (1EE6_A), and CoPEL (XP_369589).

The open reading frame of *VdPEL1* is 765 bp encoding a 255 amino acid protein. The first 20 *N*-terminal amino acids encode a signal peptide (Signal IP 4.1 server), and no transmembrane helices of VdPEL1 were found, indicating that the protein was likely to be secreted to the extracellular space. VdPEL1 has a conserved domain (39–229 amino acids) and belongs to the pectate lyase super family similar to pectate lyase A. BLAST results showed VdPEL1 homologues were present in a large number of pectate lyases of necrotrophic and hemibiotrophic plant pathogens. Phylogenetic analysis showed that all *V. dahliae* pectate lyase members were sorted into four distinct groups comprising five, two, one, and five pectate lyases each, respectively (**Figure 2**). The reference pectate lyases known to be involved in virulence and triggering defense responses in fungi and bacterial were also distributed into four branches. These results suggested that the function of pectate lyases members is significantly diverse in *V. dahliae*.

### VdPEL1 Induces Cell Death in Several Plants

**FIGURE 3:**
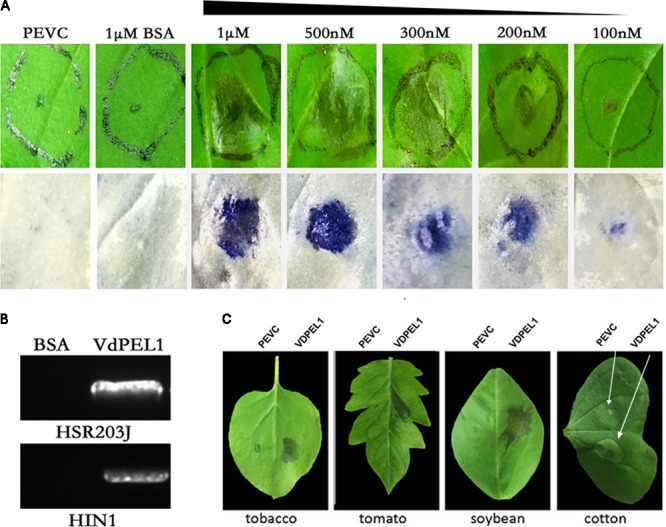
VdPEL1 induces cell death in several plants. **(A)** *N. benthamiana* leaves were infiltrated with purified VdPEL1 protein (100 nM to 1 μM), BSA (1 μM), and PEVC (*P. pastoris* culture supernatant from an empty vector control strain, purified in the same manner as VdPEL1). Two days post-infiltration, *N. benthamiana* leaves were photographed and stained with trypan blue. **(B)** *N. benthamiana* leaves were infiltrated with purified 300 nM VdPEL1 and 300 nM BSA. The expression of the *HSR203J* gene and *HIN1* gene were detected 12 h after infiltration of the recombinant VdPEL1. **(C)** Treatment of tobacco, tomato, soybean, and cotton leaves with purified 300 nM VdPEL1 and 300 nM BSA. Two days after post-infiltration, different plants leaves were photographed.

To examine the necrosis-inducing activity of VdPEL1 in *N. benthamiana*, VdPEL1 was expressed in the *P. pastoris* using the pPICZaA vector (pPICZαA: VdPEL1), which could secrete proteins into the culture media. Purified VdPEL1, with a size of 29 kDa (**Supplementary Figure S4**), was infiltrated into the mesophyll of *N. benthamiana* leaves using a syringe with different concentrations from 100 nM to 1 μM. The necrosis area occurred and increased with increasing concentrations of VdPEL1 after infiltration for 2 days compared with bovine serum albumin (BSA) or PEVC (*P. pastoris* culture supernatant from an empty vector control strain, purified in the same manner as VDPEL1), which had no HR response at 1 μM and even at 10 μM (**Figure 3A**). In parallel, the *HSR203J* gene and *HIN1* genes, which are described for HR-marker genes in tobacco plants (Takahashi et al., 2004), were significantly induced expression in VdPEL1 treated leaves (**Figure 3B**), suggesting that VdPEL1 can trigger a severe HR. To examine the host specificity of VdPEL1, we infiltrated VdPEL1 (300 nM) into the leaves of various plant species. The results demonstrated that VdPEL1 induced localized cell death in tomato, soybean (Glycine max), and cotton (*Gossypium hirsutum*) plants (**Figure 3C**). Therefore, VdPEL1 had a necrosis-inducing activity in diverse plant species.

### VdPEL1 Is Secreted Into the Apoplast in Order to Induce Cell Death in *N. benthamiana*

**FIGURE 4:**
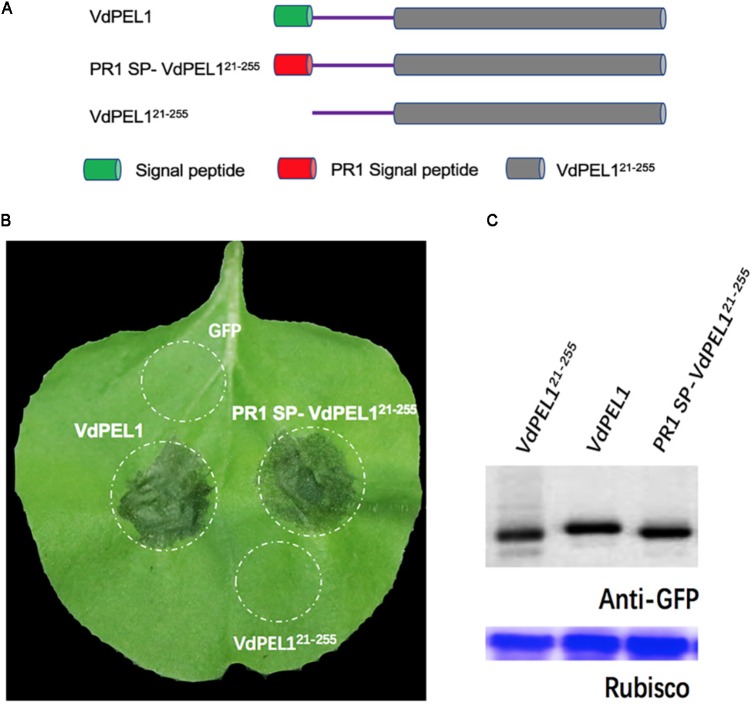
VdPEL1 is secreted into the apoplast in order to induce cell death. **(A)** Schematic presentation of the constructs examined. VdPEL1 (complete protein with the N-terminal signal peptide), VdPEL1^21-255^ (deleted the N-terminal signal peptide), and PR1 SP-VdPEL1^21-255^ (the native signal peptide replaced by the signal peptide from pathogenesis-related protein 1, PR1). **(B)** Cell death induction was detected in *N. benthamiana* leaves 5 days after infiltration with the various *A. tumefaciens* strains examined. **(C)** Immunoblot analysis of proteins from *N. benthamiana* leaves transiently expressing the examined proteins from a pYBA1132 vector.

Bioinformatic analysis showed that VdPEL1 has a signal peptide with 20 amino acids and no transmembrane helices, implying that VdPEL1 was likely to be an extracellular protein. To test whether VdPEL1 was localized to the plant apoplast to induce cell death, we constructed three *A. tumefaciens* strains: VdPEL1^21-255^ (deleted the N-terminal signal peptide), VdPEL1 (complete protein with the N-terminal signal peptide), and PR1 SP-VdPEL1^21-255^ (replaced the signal peptide from PR1) (**Figure 4A**). All the strains were infiltrated into *N. benthamiana* leaves. As expected, VdPEL1 and PR1 SP-VdPEL1^21-255^ were secreted from the plant cells to the apoplast and developed the typical necrosis, whereas expression of VdPEL1^21-255^ (remaining inside the cell) and green fluorescent protein (GFP as a negative control) didn’t appear to cause a cell death response (**Figure 4B**). Western blot assays showed that the accumulation of all the examined proteins was similar in *N. benthamiana* 5 days post-infiltration (**Figure 4C**). These results indicated that VdPEL1 must be secreted into the apoplast to trigger cell death.

### The Enzymatic Activity of VdPEL1 Is Required for Cell Death-Inducing Activity in *N. benthamiana*

**FIGURE 5:**
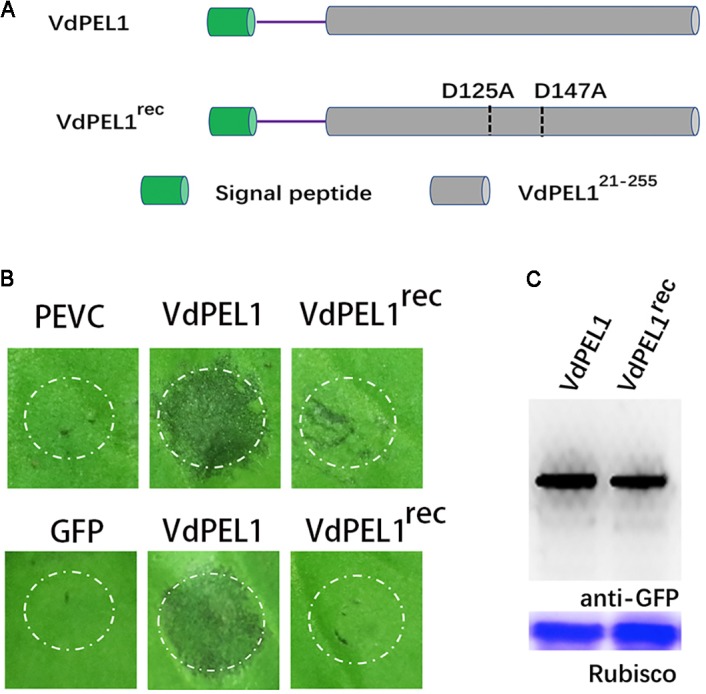
The enzymatic activity of VdPEL1 is required for cell death-inducing activity in *N. benthamiana*. **(A)** Schematic presentation of the examined constructs. VdPEL1 (the complete protein), VdPEL1^rec^ (D^125^ and D^147^ replaced with alanine, Ala). **(B)** *Upper pictures*, cell death induction was detected in *N. benthamiana* leaves 2 days after infiltration with purified VdPEL1, VdPEL1^rec^, and PEVC (*P. pastoris* culture supernatant from an empty vector control strain, purified in the same manner as VdPEL1). *Lower pictures*, cell death induction was detected in *N. benthamiana* leaves 5 days after infiltration with the examined various *A. tumefaciens* strains. **(C)** Immunoblot analysis of proteins from *N. benthamiana* leaves transiently expressing the examined proteins from a pYBA1132 vector.

Previously, pectate lyases from fungi were shown to degrade polygalacturonic acid via a β-elimination reaction (Kashyap et al., 2001). We assessed the hydrolase activity of VdPEL1 by determining the level of reducing sugar using polygalacturonic acid as substrates. We found that purified VdPEL1 had a hydrolase activity, which was affected by the temperature, Ca^2+^ concentration and pH (**Supplementary Figure S2**). Calcium (Ca^2+^) is known to be an essential co-factor for pectate lyases activity. The region around Ca^2+^ is believed to be the catalytic center site, especially aspartic acids, which are present in diverse members of the pectate lyase family (Heffron et al., 1995). As shown in **Supplementary Figure S3**, VdPEL1 contained two corresponding catalytic residues (D^125^ and D^147^) by sequence alignment. To examine the relationship between the enzymatic activity and cell death-inducing activity of VdPEL1, we replaced D^125^ and D^147^ with alanine (Ala) residues using site-directed mutagenesis and expressed the mutant proteins (VdPEL1^rec^) in *P. pastoris* (**Figure 5A** and **Supplementary Figure S4**). A hydrolase assay showed that pectate lyase activity of VdPEL1^rec^ was abolished (**Supplementary Table S1**). In addition, purified VdPEL1 induced visible cell death in *N. benthamiana*, at a much higher level than the cell death symptoms after infiltration with VdPEL1^rec^ (**Figure 5B**). We transiently expressed the wild type (VdPEL1) and mutant (VdPEL1^rec^) proteins in *N. benthamiana*. As expected, VdPEL1 triggered the cell death symptoms, while VdPEL1^rec^ and GFP lacked the ability to produce necrosis at 5 days after agroinfiltration (**Figure 5B**). Two proteins in *N. benthamiana* were confirmed using immunoblot analysis (**Figure 5C**). These results suggested that the enzymatic activity of VdPEL1 is required for cell death-inducing activity in *N. benthamiana*.

### VdPEL1 Triggers Defense Responses and Systemic Resistance in *N. benthamiana* and Cotton

**FIGURE 6:**
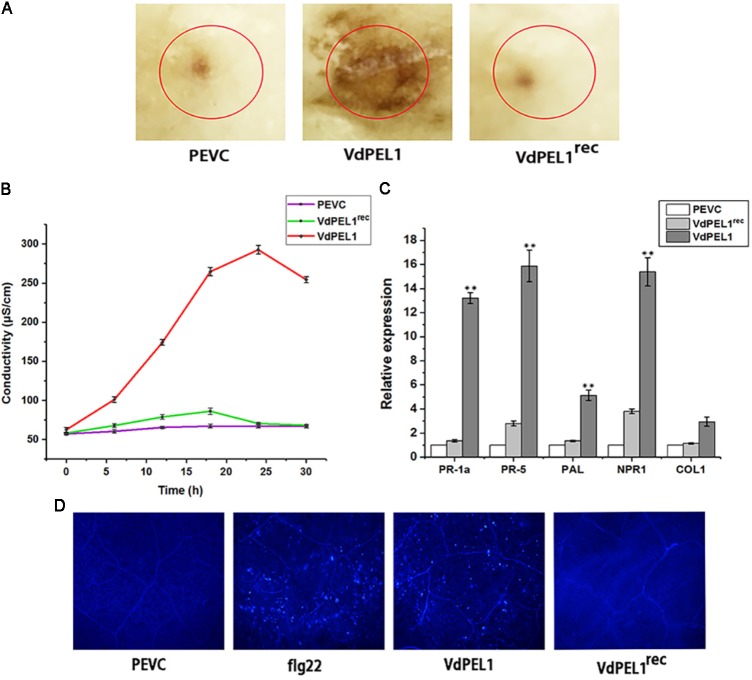
VdPEL1 induces defense responses in *N. benthamiana*. **(A)** ROS accumulation was detected in *N. benthamiana* leaves 12 h after infiltration of 300 nM purified VdPEL1, VdPEL1^rec^, and PEVC. The treated leaves were stained with DAB. **(B)** *N. benthamiana* leaves were infiltrated with 300 nM purified VdPEL1, VdPEL1^rec^, and PEVC. The conductivity was measured at the indicated time points. Error bars represent standard errors. **(C)** The transcripts of five defense response genes were measured in *N. benthamiana* leaves 24 h after infiltration of 300 nM purified VdPEL1, VdPEL1^rec^, and PEVC. qRT-PCR was performed. Error bars represent standard deviation of three independent replicates. Student’s *t*-test was performed to determine the significant differences between VdPEL1 and PEVC. *Asterisks* “^∗∗^” indicate statistically significant differences at a *p*-value < 0.01. **(D)** Callose deposition in *N. benthamiana* leaves was detected 2 days after infiltration of 300 nM flg22, purified VdPEL1, VdPEL1^rec^, and PEVC; the treated leaves were stained with aniline blue.

Plant immune system recognizes many cell death inducing proteins and activates host PTI, brings a series of typical characteristics such as accumulation of ROS, leakage of ion electrolytes, expression of defense genes, and callose deposition (Brutus et al., 2010; Zhang et al., 2014). To probe whether VdPEL1 induced immunity response, we first detected the accumulation of ROS. We observed intense staining in tobacco leaves treated with VdPEL1 (300 nM), whereas no DAB signal was detected in VdPEL1^rec^ and PEVC treated leaves (**Figure 6A**). Meanwhile, VdPEL1 also induced electrolyte leakage and displayed an increase in conductivity over time, while VdPEL1^rec^ or PEVC exhibited barely any change at the same concentration (**Figure 6B**). In addition, we examined the transcriptional induction of defense-responsive genes *PR-1a* and *PR-5*, which are involved in the SA-dependent defense pathway, *PAL* (phenylalanine ammonia lyase), *NPR1* (the nonexpressor of PR1), and *COI1* (CORONATINE INSENSITIVE 1), which is JA-responsive. As expected, the transcript levels of these defense genes were significantly up-regulated in *N. benthamiana* 24 h after treatment with VdPEL1 (**Figure 6C**). And VdPEL1^rec^ induced a slight up-regulation of defense genes (*NPR1* and *PR-5*) expression. Callose deposition was detected by aniline blue staining 24 h after VdPEL1, VdPEL1^rec^, PEVC, or flg22 treatment. *N. benthamiana* leaves inoculated with VdPEL1 or flg22 exhibited strong callose deposition compared with those inoculated with VdPEL1^rec^ or PEVC, all of which exhibited low or undetectable levels of callose deposition (**Figure 6D**).

**FIGURE 7:**
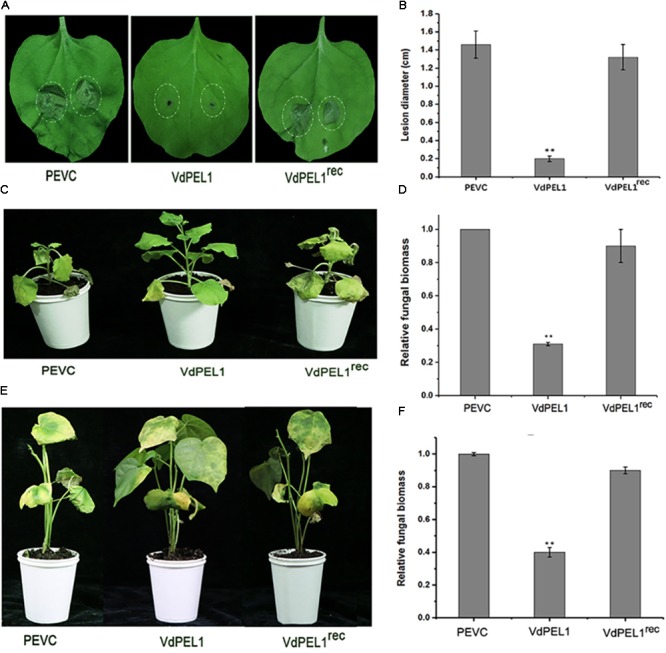
VdPEL1 confers disease resistance in *N. benthamiana* and cotton plants. **(A)** *N. benthamiana* leaves were pre-treated with 300 nM purified VdPEL1, VdPEL1^rec^, and PEVC. The treated leaves were inoculated with 5 μl of 2 × 10^6^ conidia/ml *Botrytis cinerea*. Lesions symptoms were observed and photographed at 2 days post-inoculation. **(B)** Lesion diameter of *B. cinerea* on *N. benthamiana* leaves was measured after 2 days post-inoculation. The average lesion diameter on six leaves from six plants each was determined. Error bars represent standard deviation of three independent replicates. Student’s *t*-test was performed to determine the significant differences between VdPEL1 and PEVC. *Asterisks* “^∗∗^” indicate statistically significant differences at a *p*-value < 0.01. **(C)** *N. benthamiana* leaves were pre-treated with 300 nM purified VdPEL1, VdPEL1^rec^, and PEVC and inoculated 24 h later with 1 × 10^6^ conidia/ml *V. dahliae*. The phenotypes were observed and photographed at 12 days post-inoculation. **(D)** The fungal biomasses on the *N. benthamiana* plants were determined using qRT-PCR. Error bars represent standard deviation of three independent replicates. Student’s *t*-test was performed to determine the significant differences between VdPEL1 and PEVC. *Asterisks* “^∗∗^” indicate statistically significant differences at a *p*-value <0.01. **(E)** Cotton leaves were pre-treated with 300 nM purified VdPEL1, VdPEL1^rec^, and PEVC and inoculated 24 h later with 1 × 10^6^ conidia/ml *V. dahliae*. The phenotypes were observed and photographed at 21 days post-inoculation. **(F)** The fungal biomasses on cotton plants were determined using qRT-PCR. Error bars represent standard deviation of three independent replicates. Student’s *t*-test was performed to determine the significant differences between VdPEL1 and PEVC. *Asterisks* “^∗∗^” indicate statistically significant differences at a *p*-value <0.01.

Furthermore, to explore whether VdPEL1 conferred plants disease resistance, *N. benthamiana* leaves were pretreated with 300 nM recombinant protein VdPEL1, VdPEL1^rec^ or PEVC, and 24 h later, they were inoculated with *B. cinerea* spore suspension. Leaves pretreatment with VdPEL1 restricted the development of *B. cinerea* infection, but leaves pretreatment with VdPEL1^rec^ or PEVC displayed obvious lesion (**Figure 7A**). In addition, a histogram showed determination of *B. cinerea* lesion diameter (**Figure 7B**). In parallel, tobacco and cotton plants pretreatment with VdPEL1 had more resistance to *V. dahliae*, and significantly fewer verticillium wilt symptoms and fungal biomass compared with the plants treated with VdPEL1^rec^ or PEVC controls (**Figures 7C–F**). These results collectively suggested that VdPEL1 had the capacity to trigger plant defense responses and confer disease resistance in *N. benthamiana* and cotton.

### VdPEL1 Contributes to the Pathogenicity of *V. dahliae*

**FIGURE 8:**
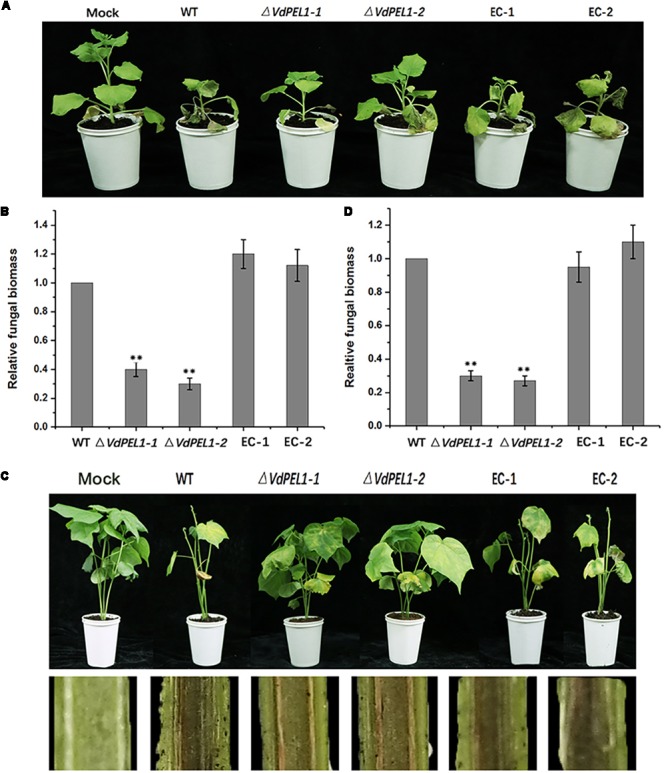
Detection of the virulence function of VdPEL1 during *N. benthamiana* and cotton pathogenesis. **(A)** Phenotypes of the inoculated *N. benthamiana* plants. Four-week-old seedlings of *N. benthamiana* were inoculated with sterile water (Mock), wild-type *Verticillium dahliae* (WT), VdPEL1 gene deletion strains (Δ*VdPEL1-1* and Δ*VdPEL1-2*), and the complementary transformants (EC-1 and EC-2). The virulence phenotypes were photographed 12 days post-inoculation. **(B)** The fungal biomass quantification of WT, Δ*VdPEL1-1* and Δ*VdPEL1-2*, and EC-1 and EC-2 inoculated on *N. benthamiana* was determined using qPCR. Error bars represent standard deviation of three independent replicates. Student’s *t*-test was performed to determine the significant differences between mutants and WT stain. *Asterisks* “^∗∗^” indicate statistically significant differences at a *p*-value < 0.01. **(C)** Phenotypes of inoculated cotton plants. Two-week-old seedlings of cotton were inoculated with sterile water (Mock), wild-type *Verticillium dahliae* (WT), VdPEL1 gene deletion strains (Δ*VdPEL1-1* and Δ*VdPEL1-2*), and the complementary transformants (EC-1 and EC-2). The disease symptoms 21 days after inoculation are shown at the *top*, and the discoloration of the inoculation shoot longitudinal sections is shown at the *bottom*. **(D)** The fungal biomass quantification of WT, Δ*VdPEL1-1* and Δ*VdPEL1-2*, and EC-1 and EC-2 inoculated on cotton plants were determined using qPCR. Error bars represent standard deviation of three independent replicates. Student’s *t*-test was performed to determine the significant differences between mutants and WT stain. *Asterisks* “^∗∗^” indicate statistically significant differences at a *p*-value < 0.01.

Pectate lyases are generally involved in the pathogenicity of fungi (Collmer, 1986). To determine the possible contribution of VdPEL1 to *V. dahliae* virulence, we assessed the expression patterns of *VdPEL1* during different stages of post-inoculation. *VdPEL1* was strongly expressed in tobacco and cotton at 1.5–3 days after inoculation, and then sharply declined from 3 days onward (**Supplementary Figure S5**). Next, we generated two independent *VdPEL1* deletion lines (Δ*VdPEL1-1* and Δ*VdPEL1-2*) and the complementary transformants (EC-1 and EC-2) by reintroducing the *VdPEL1* gene (**Supplementary Figure S6**). All of the strains examined showed normal development, and there was no influence on radial growth and colony morphology (**Supplementary Figure S7**). To investigate the contribution of VdPEL1 to *V. dahliae* virulence, the wild type *V. dahliae* and *VdPEL1* mutant strains were inoculated onto *N. benthamiana* plants and cotton plants. Interestingly, *VdPEL1* deletion strains displayed significantly reduced virulence on tobacco plants compared with the wild-type *V. dahliae*. EC-1 and EC-2 recovered the high virulence phenotypes (**Figure 8A**). Similar results were observed in cotton plants 21 days after inoculation with all transformants. Δ*VdPEL1-1* and Δ*VdPEL1-2* decreased disease susceptibility, whereas EC-1 and EC-2 and wild strains caused more symptoms of necrosis, wilting, and vascular discoloration (**Figure 8C**). These results indicated that VdPEL1 played a positive role in *V. dahliae* virulence. This conclusion was further supported by the observation that the fungal biomass of *VdPEL1* deletion lines was significantly lower than the biomass of the wild-type and complementary transformants in inoculated *N. benthamiana* and cotton plants (**Figures 8B,D**). These results confirmed that VdPEL1 contributed to virulence to *V. dahliae*.

## Discussion

The plant exocyst has recently emerged as an important battleground in plant–pathogen interactions (Žárský et al., 2013; Fujisaki et al., 2015). The plant cell wall serves as a natural barrier to limit the invasion of pathogens. To penetrate and colonize plants, phytopathogenic fungi produce a diverse group of plant CWDEs, which are involved in the generation of plant diseases and pathogenesis (King et al., 2011; Glass et al., 2013; Kubicek et al., 2014). Among the CWDEs, the pectate lyases are examined more closely because of their crucial roles in degrading plant pectin, which exists widely in plant cell walls and cell linings to maintain cell wall integrity. In this study, we identified a secreted pectate lyase VdPEL1 from *V. dahliae* culture supernatant, which has the ability to trigger immunity plant responses and contributes to *V. dahliae* virulence. In addition, our study also found that the enzymatic activity of VdPEL1 was necessary for induced cell death and PTI responses. Our data provide a new avenue to advance the understanding of host–pathogen interactions.

*Verticillium dahliae*, a soil-borne hemibiotrophic pathogen, attacks the plant roots and spreads to the leaves through the xylem vessels resulting in verticillium wilt of cotton and diseases in over 400 different plant species (Larsen et al., 2007; Vallad and Subbarao, 2008). Recent reports demonstrated that *V. dahliae* secreted a large amount of pectate lyases to catalyze the degradation of the pectin and facilitate penetration during its infection processes (Bartnicki-Garcia, 1968; Durrands and Cooper, 1988a,b; Pietro et al., 2009; Tzima et al., 2010). Although pectate lyases are particularly abundant and evolutionary preserved, phylogenetic analysis showed that the pectate lyases of *V. dahliae* are divided into four groups. According to the difference of virulence and defense responses in fungi and bacteria, pectate lyases were also distributed into four branches (**Figure 2**). We hypothesized that the pectate lyases play diverse functions in pathogenesis and confer the ability of *V. dahliae* to cause disease on such a broad host range.

The HR, a form of plant cell death in the tissues surrounding the lesion, is regarded as a plant defense response to block pathogen infection (Glazebrook, 2005; Jones and Dangl, 2006; Yamada et al., 2016). The ability to recognize a few nanograms of purified VdPEL1 resulting in rapid leaf tissue necrosis was observed in soybean, tomato, cotton, and *N. benthamiana* (**Figure 3C**). In addition, the range of plant species responding to VdPEL1 may be larger than we detected. We confirmed that VdPEL1 triggered defense responses, including the accumulation of ROS, leakage of ion electrolytes, deposition of callose, and expression of defense genes (**Figure 6**).

As we know, due to the diversity of the host and the inability of fungicides to affect the pathogen once in the plant vascular system, verticillium wilt diseases are difficult to control. The most sustainable manner to control these diseases is the use of resistant cultivars. Thus, it is relevant to identify the new PAMPs or DAMPs, which can provide materials for disease-resistant breeding. VdPEL1 could induce plant immunity and has the potential to be used in plant breeding and as a biological pesticide.

Previous studies showed that many fungal CWDEs, including xyloglucanases, glucanases, and cellulases, can trigger cell-death responses independent of their enzymatic activity (Ma et al., 2014, 2015; Gui Y.-J. et al., 2017; Zhu et al., 2017a). Endopolygalacturonase 1 (EG1) has two biological activities (enzymatic activity and elicitor activity) that are independent of each other in *B. cinerea* (Poinssot et al., 2003). However, an extracellular cutinase isolated from *V. dahliae* triggered plant defense responses required the enzymatic activity in *N. benthamiana* (Gui Y. et al., 2017). To test whether the enzymatic activity of VdPEL1 was required to trigger the plant immune responses, the site-directed mutagenesis of two residues in a conserved motif of VdPEL1 resulted in the loss of enzymatic activity. In contrast to VdPEL1, we surprisingly found that VdPEL1^rec^ resulted in the loss of the cell death-inducing activity and the function of a series of plant defense responses and systemic resistance (**Figures 6, 7**). These results indicated that the enzymatic activity of VdPEL1 was necessary to trigger defense responses in *N. benthamiana* and cotton plants.

The pectate lyases have been implicated in pathogenicity and virulence in several plant pathogens. For example, in *C. coccodes*, the pectate lyase gene *CcpelA* contributes virulence on tomato, and in *C. gleosporoides*, deletion of the pectate lyase gene *PelB* resulted in a substantial loss of virulence on avocado (*Persea americana*) fruit (Yakoby et al., 2001; Ben-Daniel et al., 2011). Not all fungal pectate lyases have been conclusively shown to be involved in pathogenicity and virulence. For example, *PelA* (a pectate lyase gene form *Fusarium graminearum*) knock-out strains did not show attenuated virulence during the infection of wheat coleoptiles (Blum, 2017). The expression of *VdPEL1* was most significantly up-regulated during the infection stage (1.5–3 days) before dropping sharply to the initial level (**Supplementary Figure S5**). The very strong and early expression of *VdPEL1* may help the pathogen to extract nutrition, in addition to the more obvious role of physically facilitating invasion of the host tissue. Meanwhile, we observed that targeted *VdPEL1* deletion resulted in significantly compromised virulence of Vd991 on tobacco and cotton plants (**Figure 8**).

Unlike effectors, which interferes with the plant defense response leading to ETS, VdPEL1 appeared to be a major virulence factor due to its enzymatic function, similar to VdCUT11 (Gui Y.-J. et al., 2017). It may indicate that VdPEL1 executes the pectate lyase activity to degrade pectin in the roots, which results in the invasion of the pathogens and the release of plant cell wall fragments (DAMPs). We speculated that the immunity triggered by VdPEL1 was likely to be mediated by the degradation of plant cell wall polymers that release pectin hydrolysis products (DAMPs), which in turn, trigger defense responses in plants.

The plant cell wall may be damaged by abiotic assaults or biotic assaults, resulting in the tissue or cellular damage, which is perceived as danger signals that function as DAMPs (Bianchi, 2006; Choi and Klessig, 2016). Generally, DAMPs appear in the apoplast and induce innate immune responses. For instance, cutin monomers and plant elicitor peptides (Peps), which are produced by pathogens depolymerization, can act as DAMPs (Chassot and Métraux, 2005; Huffaker et al., 2006; Yamaguchi et al., 2006). Similarly, OGs, fragments of the pectic polysaccharide homogalacturonan, can be released by pathogen-encoded hydrolytic enzymes to induce innate immune responses, including MAPK activation, callose deposition, ROS production and defense gene up-regulation (Decreux and Messiaen, 2005; Denoux et al., 2008).

Successful pathogens deliver effectors to surmount the host PTI response and establish infection (Jones and Dangl, 2006; Gimenez-Ibanez et al., 2009). For example, a RXLR effector suppressed XEG1-triggered immunity in oomycetes (Ma et al., 2015). In *V. dahliae*, carbohydrate-binding modules (CBMs) act as effectors, suppressing the GH12 protein and VdCUT11-triggered immunity and thus, facilitating host colonization (Gui Y. et al., 2017; Gui Y.-J. et al., 2017). Whether effectors mediate the suppression of VdPEL1 merits further investigation.

## Author Contributions

YD and DQ designed the experiments. YY performed most of the experiments and wrote the paper. XY and BL participated in some part of the study.

## Conflict of Interest Statement

The authors declare that the research was conducted in the absence of any commercial or financial relationships that could be construed as a potential conflict of interest.

## Supplementary Material

FIGURE S1 The MS/MS sequencing information. Proteins corresponding to Peak A were digested, and the peptides generated were analyzed using mass spectrometry. The detected peptides were covered with *yellow color* and matched against a protein from *V. dahliae*.

FIGURE S2 The analysis of the hydrolase activity of VdPEL1 with different temperatures, Ca^2+^ concentrations or pH. The reduced sugars were quantified using a standard calibration curve obtained with polygalacturonic acid. All the experiments were replicated three times. Standard errors from three biological replicates are shown.

FIGURE S3 Sequence alignment of the *V. dahliae* pectate lyase family proteins. Sequence alignment of all the *V. dahliae* pectate lyase family proteins and known cutinases from other fungi. The accession numbers of known pectate lyases from other fungi and bacteria are: 1EE6 (a pectate lyase from *Bacillus* sp. strain Ksm-P15), Pel A (pectate lyase A from *Erwinia chrysanthemi*). Two *red triangles* indicated possible catalytic residues of VdPEL1 (D^125^ and D^147^).

FIGURE S4 SDS-PAGE of VdPEL1 and VdPEL1^rec^ recombinant proteins. VdPEL1 is the native protein; VdPEL1^rec^ is a site-directed mutagenized protein, in which D^125^ and D^147^ were substituted with Ala. Two recombinant proteins were stained with Coomassie blue.

FIGURE S5 *VdPEL1* expression analysis during infection of *N. benthamiana* and cotton roots. **(A)** The expression analysis of VdPEL1 in cotton roots. **(B)** The expression analysis of VdPEL1 in tobacco roots. The control (C) was mixed with non-inoculated conidia and cotton or tobacco root tissue. The housekeeping gene *β-tubulin* (VDAG_10074) was used as an endogenous control. Error bars represent standard errors.

FIGURE S6 Schematic view of the targeted deletion of *VdPEL1* in *V. dahliae*. Two flanking sequences of the target gene and hygromycin resistance cassette were constructed into a fusion fragment. The fusion amplicon was integrated into the pGKO2-gateway vector using a homologous recombination method. The vectors were transferred into the *Agrobacterium tumefaciens* AGL-1 strain for fungal transformation with the wild-type (WT) strain Vd991. Two test primers were used to identify the fusion amplicon and positive targeted gene-deletion stains using PCR, respectively.

FIGURE S7 The targeted deletion of *VdPEL1* does not affect radial growth and colony morphology. **(A)** The radial growth and colony morphology of the wild-type *V. dahliae* (WT), two VdPEL1 deletion strains (ΔVdPEL1-1 and ΔVdPEL1-2), and two ectopic transformants (EC-1 and EC-2) were determined after 11 days of incubation on PDA media at 25°C. **(B)** Colony diameters were determined at the time points indicated. Values shown are the average of three colony diameters. Error bars represent standard deviations.

TABLE S1 Hydrolysis activity test.

TABLE S2 Primers used in this study.
