# Supplementary figures and images for: A Verticillium dahliae Pectate Lyase Induces Plant Immune Responses and Contributes to Virulence
Source: Front Plant Sci. 2018 Sep 13;9:1271. doi: 10.3389/fpls.2018.01271 (PMC6146025; doi:10.3389/fpls.2018.01271)

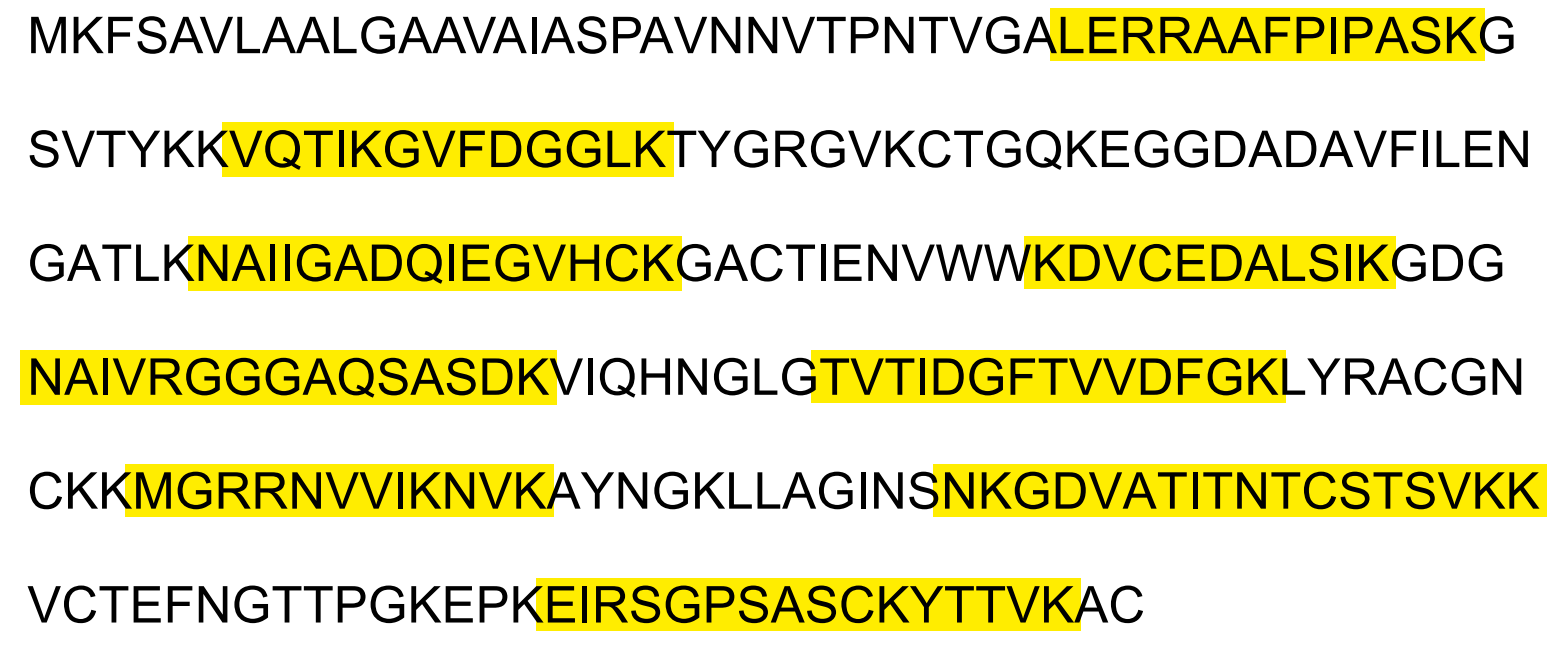

Supplement: FIGURE S1 — The MS/MS sequencing information. Proteins corresponding to Peak A were digested, and the peptides generated were analyzed using mass spectrometry. The detected peptides were covered with yellow color and matched against a protein from V. dahliae. [file Image_1.TIF]

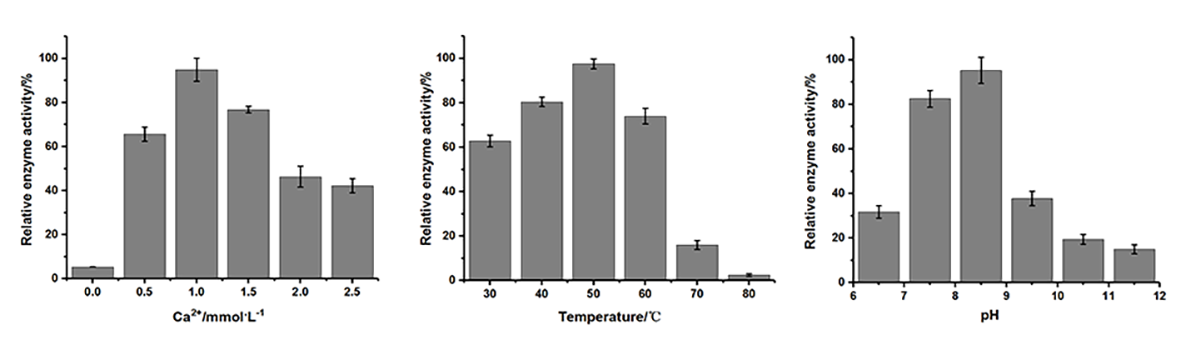

Supplement: FIGURE S2 — The analysis of the hydrolase activity of VdPEL1 with different temperatures, Ca2+ concentrations or pH. The reduced sugars were quantified using a standard calibration curve obtained with polygalacturonic acid. All the experiments were replicated three times. Standard errors from three biological replicates are shown. [file Image_2.TIF]

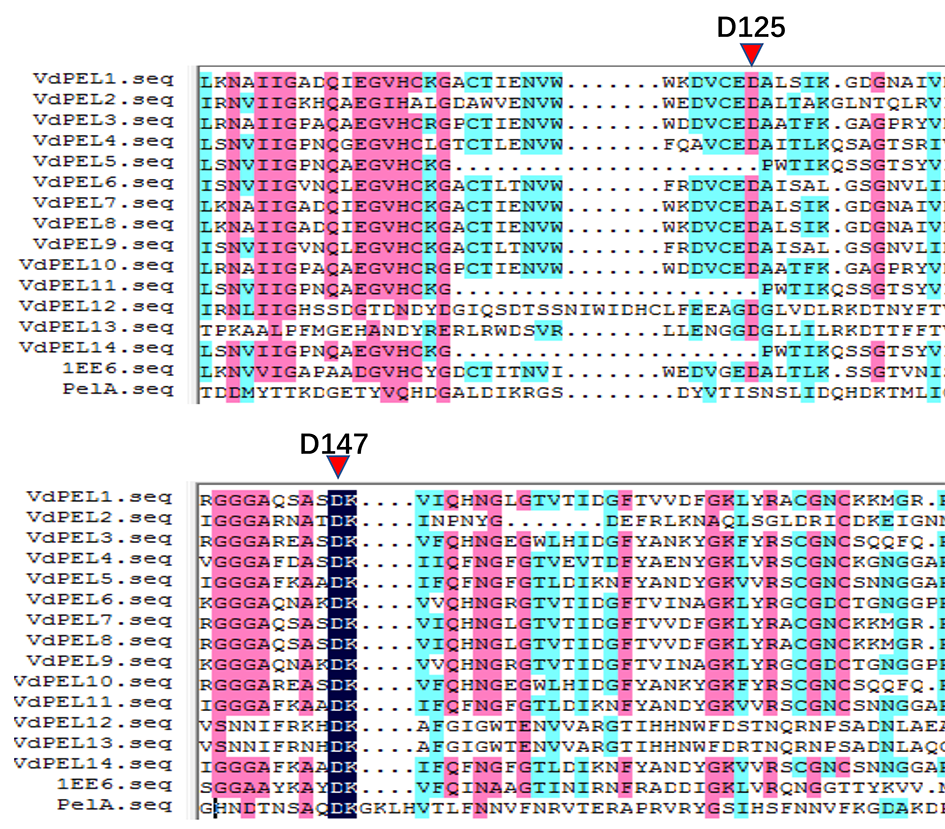

Supplement: FIGURE S3 — Sequence alignment of the V. dahliae pectate lyase family proteins. Sequence alignment of all the V. dahliae pectate lyase family proteins and known cutinases from other fungi. The accession numbers of known pectate lyases from other fungi and bacteria are: 1EE6 (a pectate lyase from Bacillus sp. strain Ksm-P15), Pel A (pectate lyase A from Erwinia chrysanthemi). Two red triangles indicated possible catalytic residues of VdPEL1 (D125 and D147). [file Image_3.TIF]

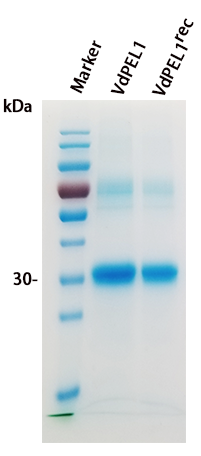

Supplement: FIGURE S4 — SDS-PAGE of VdPEL1 and VdPEL1rec recombinant proteins. VdPEL1 is the native protein; VdPEL1rec is a site-directed mutagenized protein, in which D125 and D147 were substituted with Ala. Two recombinant proteins were stained with Coomassie blue. [file Image_4.TIF]

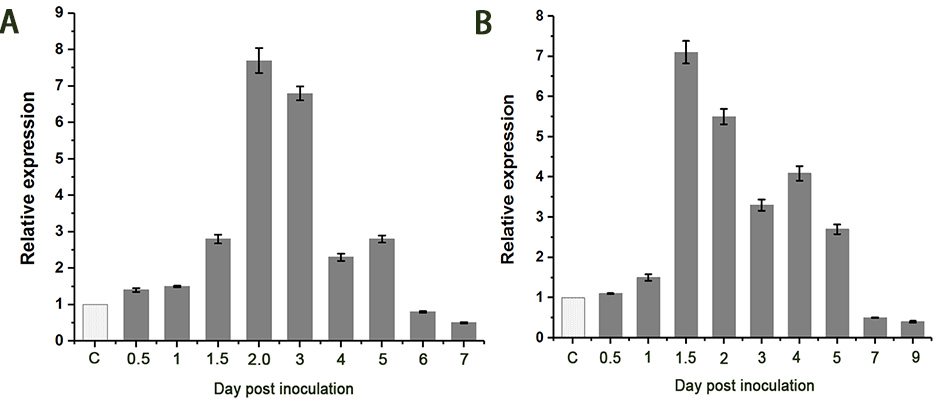

Supplement: FIGURE S5 — VdPEL1 expression analysis during infection of N. benthamiana and cotton roots. (A) The expression analysis of VdPEL1 in cotton roots. (B) The expression analysis of VdPEL1 in tobacco roots. The control (C) was mixed with non-inoculated conidia and cotton or tobacco root tissue. The housekeeping gene β-tubulin (VDAG_10074) was used as an endogenous control. Error bars represent standard errors. [file Image_5.TIF]

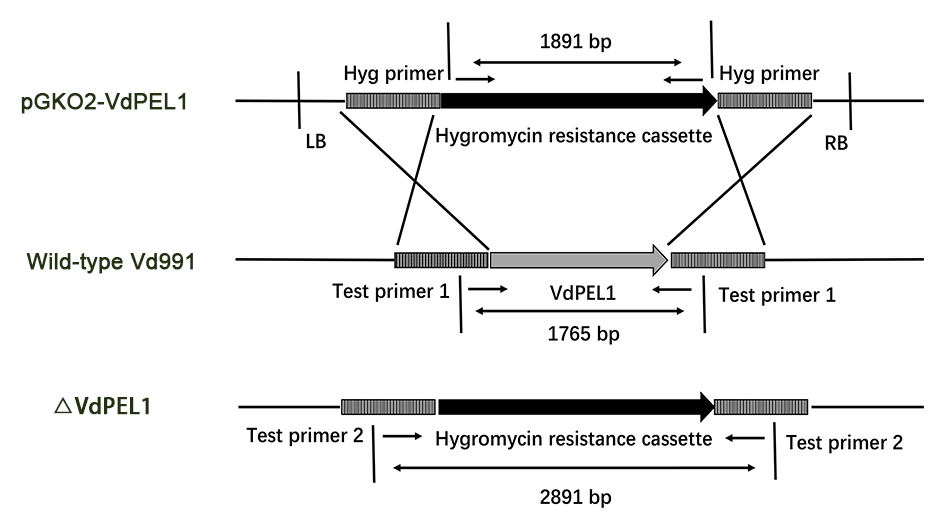

Supplement: FIGURE S6 — Schematic view of the targeted deletion of VdPEL1 in V. dahliae. Two flanking sequences of the target gene and hygromycin resistance cassette were constructed into a fusion fragment. The fusion amplicon was integrated into the pGKO2-gateway vector using a homologous recombination method. The vectors were transferred into the Agrobacterium tumefaciens AGL-1 strain for fungal transformation with the wild-type (WT) strain Vd991. Two test primers were used to identify the fusion amplicon and positive targeted gene-deletion stains using PCR, respectively. [file Image_6.TIF]

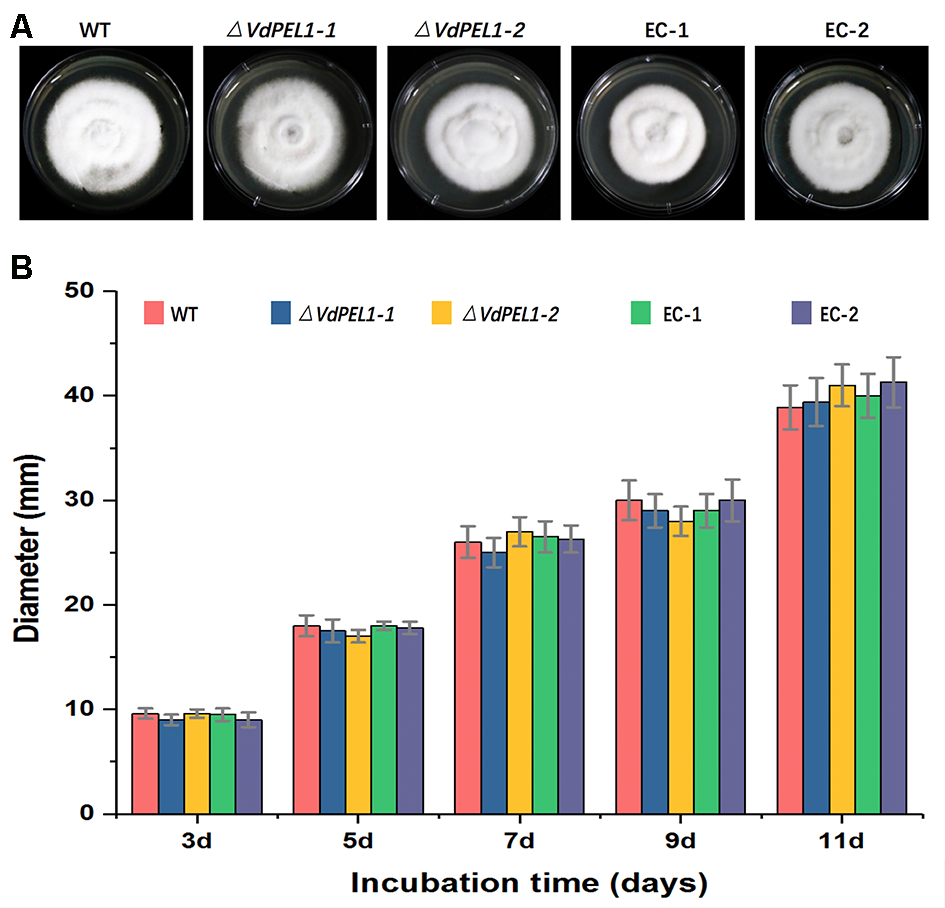

Supplement: FIGURE S7 — The targeted deletion of VdPEL1 does not affect radial growth and colony morphology. (A) The radial growth and colony morphology of the wild-type V. dahliae (WT), two VdPEL1 deletion strains (ΔVdPEL1-1 and ΔVdPEL1-2), and two ectopic transformants (EC-1 and EC-2) were determined after 11 days of incubation on PDA media at 25°C. (B) Colony diameters were determined at the time points indicated. Values shown are the average of three colony diameters. Error bars represent standard deviations. [file Image_7.tif]
