# Supplementary material for: A Verticillium dahliae Pectate Lyase Induces Plant Immune Responses and Contributes to Virulence
Source: Front Plant Sci. 2018 Sep 13;9:1271. doi: 10.3389/fpls.2018.01271 (PMC6146025; doi:10.3389/fpls.2018.01271)
Supplement: TABLE S1 — Hydrolysis activity test. [file Table_1.PDF]

Table 1. Hydrolysis Activity of VdPEL1 and VdPEL1^{rec} toward polygalacturonic acid

Protein	Units/mg	(%)
VdPEL1	9.98±0.32	100±5.4
VdPEL1^{rec}	0.23±0.09	2.3±0.43

Two protein independent measurements with three replicates each. Standard errors are shown.
