# Supplementary material for: A Verticillium dahliae Pectate Lyase Induces Plant Immune Responses and Contributes to Virulence
Source: Front Plant Sci. 2018 Sep 13;9:1271. doi: 10.3389/fpls.2018.01271 (PMC6146025; doi:10.3389/fpls.2018.01271)
Supplement: TABLE S2 — Primers used in this study. [file Data_Sheet_1.PDF]

Table S2. Primers used in this study

Primer name	Primer 5'-3	Purpose
VdPEL1 F	CGGGATCCGTCAACAACGTCACCCCCAA	To amplify the DNA fragment of VdPEL1 for expression in *Pichia pastoris*
VdPEL1 R	CGGAATTCGCAAGCCTTGACGGTGGTGT	To amplify the DNA fragment of VdPEL1 for expression in *Pichia pastoris*
VdPEL1rec F	TATTGTGCGCGGTGGAGGAGCCCAGTCTGCTTCTGCAAAGGTTATCCAGCA	To amplify the DNA fragment of site-directed mutagenized VdPEL1rec for expression in *Pichia pastoris*
VdPEL1rec R	CGCGCACAATAGCGTTACCATCCCCCT TAATACTCAG CGCTGCCTCGCAGACA	To amplify the DNA fragment of site-directed mutagenized VdPEL1rec for expression in *Pichia pastoris*
VdPEL121-255-F	CGGAATCCGTCAACAACGTCACCCCCAACA	To transient expression VdPEL121-255 protein (deleted the N-terminal signal peptide)
VdPEL121-255-R	GCTCTAGAGCAAGCCTTGACGGTGGTGT	To transient expression VdPEL121-255 protein (deleted the N-terminal signal peptide)
VdPEL1-T-F	CGGAATCCATGAAGTTCTCCGCTGTCCTCGCT	To transient expression VdPEL protein (with the N-terminal signal peptide)
VdPEL1-T-R	GCTCTAGAGCAAGCCTTGACGGTGGTGTA	To transient expression VdPEL protein (with the N-terminal signal peptide)
PR1 SP-VdPEL121-255-F	CGGAATCCATGCCTCCACAAAAAGAAAACC	To transient expression PR1 SP-VdPEL121-255 protein (replaced the signal peptide from pathogenesis-related protein 1 (PR1))
PR1 SP-VdPEL121-255-R	GCTCTAGAGCAAGCCTTGACGGTGGTGTA	To transient expression PR1 SP-VdPEL121-255 protein (replaced the signal peptide from pathogenesis-related protein 1 (PR1))
RT-qPCR-NbEF1α-F	AGGATACAACCCTGACAAGA	*N. benthamiana EF-1α* gene used as qRT-PCR reference
RT-qPCR-NbEF1α-R	AGGATACAACCCTGACAAGA	*N. benthamiana EF-1α* gene used as qRT-PCR reference
RT-qPCR-EF-1α-F	TGAGTTCGAGGCTGGTATCT	*V.dahliae EF-1α* gene used for qPCR measurement of pathogen levels and as qRT-PCR reference
RT-qPCR-EF-1α-R	CACTTGGTGGTGTCCATCTT	*V.dahliae EF-1α* gene used for qPCR measurement of pathogen levels and as qRT-PCR reference
RT-qPCR-18S-F	CGGCTACCACATCCAAGGAA	Cotton 18s gene used as qRT-PCR reference
RT-qPCR-18S-R	TGTCACTACCTCCCCGTGTCA	Cotton 18s gene used as qRT-PCR reference
RT-qPCR-PR1a-F	GTGGGTCGATGAGAAACAGTAT	RT-qPCR for validation of *PR1a* gene transcription level in *N. benthamiana* leaves
RT-qPCR-PR1a-R	GAACCCTAGCACATCCAACA	RT-qPCR for validation of *PR1a* gene transcription level in *N. benthamiana* leaves
RT-qPCR-NPR1-F	GGAGCAAGCAGAAAGAAGAGA	RT-qPCR for validation of *NPR1* gene transcription level in *N. benthamiana* leaves
RT-qPCR-NPR1-R	GTTTAGCCAGGCCAACTCTAT	RT-qPCR for validation of *NPR1* gene transcription level in *N. benthamiana* leaves
RT-qPCR-PR5-F	GGGCCAATCTTGGAGCATTA	RT-qPCR for validation of *PR5* gene transcription level in *N. benthamiana* leaves
RT-qPCR-PR5-R	CAGTCTCCAGTCTCACAATTACC	RT-qPCR for validation of *PR5* gene transcription level in *N. benthamiana* leaves
RT-qPCR-COI1-F	GGCTTGACGTACTTAGGGAAATA	RT-qPCR for validation of *COI1* gene transcription level in *N. benthamiana* leaves
RT-qPCR-COI1-R	GGGACACCTTTGCAGTAAGA	RT-qPCR for validation of *COI1* gene transcription level in *N. benthamiana* leaves
RT-qPCR-PAL-F	ATTGCTGGTTTGCTCACTGG	RT-qPCR for validation of *PAL* gene transcription level in *N. benthamiana* leaves
RT-qPCR-PAL-R	TCCTTAGGCTGCAACTCGAA	RT-qPCR for validation of *PAL* gene transcription level in *N. benthamiana* leaves
